# Drug Delivery Systems for Glaucoma: A Narrative Review

**DOI:** 10.3390/ph17091163

**Published:** 2024-09-02

**Authors:** Antonio M. Fea, Veronica Vallino, Michela Cossu, Valentina Marica, Cristina Novarese, Michele Reibaldi, Francesco Petrillo

**Affiliations:** Department of Ophthalmology, “City of Health and Science” Hospital, 10126 Turin, Italy; antoniofea@icloud.com (A.M.F.); veronica.vallino@gmail.com (V.V.); michela.cossu@hotmail.com (M.C.); vale.marica.vm@gmail.com (V.M.); cristina.novarese@edu.unito.it (C.N.); mreibaldi@libero.it (M.R.)

**Keywords:** glaucoma, punctal plug delivery systems, conjunctival fornix delivery systems, drug-laden contact lenses, periocular drug delivery systems, intracameral delivery systems

## Abstract

Glaucoma is one of the world’s leading causes of blindness, and its management is challenging. The main objective is to lower intraocular pressure through medical, para-surgical, and surgical therapy. Medical therapy often represents the first line of treatment. Although effective in many cases, the eye drops are accompanied by significant problems. They require high patient compliance and can be associated with various side effects, limiting their efficacy. Consequently, the research for new drug delivery systems trying to overcome these limitations is ongoing: numerous devices are developing and gradually entering clinical practice. These new therapeutic options may offer better control of the intraocular pressure, with fewer side effects, and are less dependent on patients’ compliance. Hence, the research in this field continues to flourish. This review summarizes the most recent findings in the scientific literature, underlines the role and possible limitations of the new glaucoma drug delivery systems in clinical practice, and recognizes their new horizons and perspectives.

## 1. Introduction

Glaucoma is a leading cause of irreversible blindness worldwide and poses a significant public health problem. It has been estimated that about 80 million people are affected by this condition, and 11 million are bilaterally blind [1]. The disease consists of an optic neuropathy characterized by progressive degeneration of retinal ganglion cells, resulting in cupping (an excavated appearance of the optic disc) and visual loss [2]. The most common form of glaucoma is open-angle (OAG), and intraocular pressure (IOP) is the leading risk factor for its development and progression. Although there is no cure, glaucoma therapies intend to preserve visual function by lowering IOP to the target pressure [3]. The term target pressure refers to an IOP below which it is reasonable to estimate the rate of disease progression to be sufficiently slow to minimize the risk of further symptomatic visual loss. Available treatment options include medical therapy (both topical drops and oral medications), laser therapy, and incisional surgery. Topical medications are often the first-line treatment. Even when eye drops are adequate, patients’ adherence to daily medication is a significant challenge. Patients often require multiple medications and doses, and older people have difficulty instilling eye drops. It has been estimated that up to 80% of patients do not use their IOP-lowering eye drops as prescribed [4,5]. In glaucoma, poor medication adherence has been associated with worse visual outcomes [6]. Besides adherence problems, significant barriers to drug delivery can result in suboptimal medication levels in the target tissues. Additionally, the eye drop delivery of drugs is characterized by a pulsatile fashion with a peak drug concentration followed by a through before the next dose, which could result in worse control of the IOP and a higher risk of adverse effects [7]. Sustained release drug delivery systems can potentially avoid many of the limitations of eye drops. A continuous drug release is associated with sustained lowering of the IOP, a reduced total dose, and a lower or slower systemic exposure, which reduces the risk of systemic side effects. Moreover, eliminating the need to remember to use medication for that period and bypassing the challenges of administering them at the right time helps to overcome adherence problems [8]. Several sustained-release drug delivery systems are in preclinical development or clinical trials to treat glaucoma and ocular hypertension (OHT). This narrative review aims to provide an overview of recent advancements in drug delivery systems for glaucoma. In particular, we will focus on five innovative approaches: drug-eluting punctal plugs, periocular inserts, contact lenses, subconjunctival implants, and intracameral implants. Through a critical analysis of the existing literature, we will examine the characteristics, advantages, limitations, and future perspectives of each of these systems, with the aim of offering a comprehensive understanding of their potential clinical applications and associated challenges.

## 2. Punctal Plugs Delivery System

**Figure 1:**
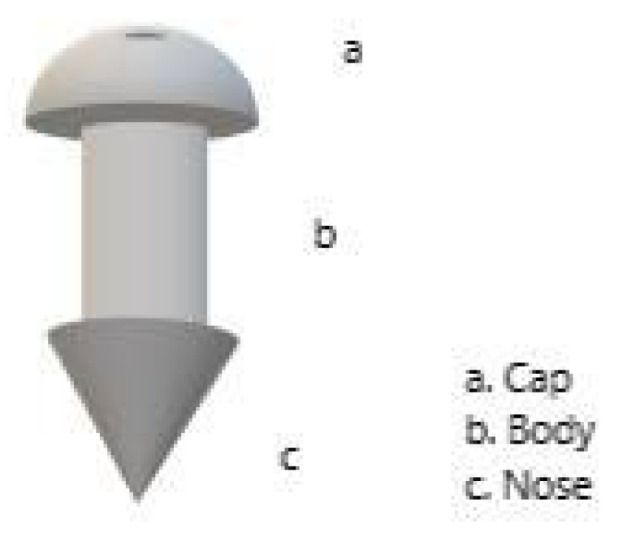
Punctal plug.

Punctal plugs (Figure 1) are rod-shaped medical devices, usually made of a polymeric material, designed to be implanted into the tear ducts. In order to reduce tear drainage and increase the tears on the ocular surface, they block the punctum and the canaliculus [9]. These plugs can be loaded with drugs that are slowly released in a unidirectional manner toward the ocular surface, prolonging contact with the eye surface. Drug mixing with the tear fluid and subsequent delivery to the eye surface and intraocular tissues is thus enhanced. Punctal plugs can be perpetual (for dry eye) or temporary (for drug delivery). Plugs come in a variety of shapes and sizes. Each part of the structure can change, such as the core (polymer matrix or drug matrix permeable to tear fluid), the cap (semi- or impermeable membrane with one or more pores), the body (impermeable to drug and tear fluid), and the nose (assists the insertion process) [7]. Inside the core of the plug is a potentially possible place to load the drug solution, suspension, emulsion, nanoparticle or microparticle, or liposome suspensions [10].

Glaucoma patients may benefit from punctal plugs for three main reasons. Ocular surface disease (OSD) frequently coexists with glaucoma and may be initiated or exacerbated by topical medications. Patients with OSD may have an aqueous deficiency and dysfunctional tear film circulation. Recent studies have underlined the utility of punctal plugs in curing these patients because they can prevent tears from draining through the nasolacrimal ducts, thereby maintaining a greater volume of tears on the surface of the eye and relieving dry eye symptoms [11,12,13]. Furthermore, punctal plugs could reduce the systemic absorption of drugs and improve their efficacy on the target tissue. Lastly, when loaded with antiglaucoma drugs, they represent an alternative to eye drops, thus reducing their burden and compliance issues.

Many studies supported the advantages of punctal plugs in patients with glaucoma (Table 1).

Chen et al. used a temporary collagen punctal plug as a pre-test before the decision on permanent punctal closure or long-term plug use for patients with dry eye disease (DED) and primary open-angle glaucoma (POAG). In order to predict the efficacy of the long-term ones and find possible complications, it was used a non-expensive temporary collagen plug. It has been demonstrated that in eyes responding to temporary plugs with fewer DED symptoms and reduced IOP without complications, a long-term punctal plug or permanent punctal closure could be considered. Using punctal occlusion can be safer because it decreases the absorption of glaucoma medications into nasal mucosae, such as beta-blockers, preventing heart rate disturbances [14]. 

Sherwin et al., in a randomized controlled study of punctal plug placement in glaucoma patients treated with a prostaglandin analog, found reduced OSDI scores, increased tear break-up time, reduced corneal staining, and decreased tear osmolarity. Curiously, IOP also decreased, probably because of increased holding and assimilation of prostaglandin [15]. Zimmermann et al. demonstrated that nasolacrimal occlusion is a simple technique that increases the ocular bioavailability of topically applied ocular drugs and reduces the probability of adverse systemic effects. Punctal occlusion can reduce systemic drug absorption of topical timolol by more than 60%. This technique seems safe, simple, and effective [18]. Also, Huang et al. found, after punctal plug occlusion, a statistically significant reduction in IOP with a variance of 1.82 mm Hg or 10.7% (baseline of 16.96 mm Hg) compared with before punctal occlusion [19].

Punctal plugs can be filled with medication and act as a conduit for its delivery. However, their capacity is restricted to accommodating low doses of medication, making corticosteroids and prostaglandins the primary drugs suitable for this purpose due to their effectiveness at low concentrations. The sole commercially available plug system is Dextenza™, specifically designed to administer dexamethasone for the management of post-operative inflammation and pain. Nevertheless, several research efforts have been directed towards developing systems for prostaglandin delivery as well [20,21].

Perera et al. developed travoprost tear plugs named OTX-TP (Ocular Therapeutix, Inc., Bedford, MA, USA) utilizing polylactic acid particles enclosed in polyethylene glycol absorbable hydrogel rods. These microparticles hydrolyze to deliver travoprost over a 90-day period. In their initial single-arm study, all plugs were directly visible post-insertion without slit lamp examination. Retention rates, assessed through visualization, remained at 100% for the first 10 days, declining to 42% by day 30 [16]. In a subsequent phase 2 trial, patients were divided into two groups and randomly prescribed to receive either two times a day artificial tears or timolol 0.5% twice daily with a drug-free punctal plug. Both groups showed clinically significant reductions in IOP from baseline, with the OTX-TP group demonstrating a reduction of 4.5–5.7 mm Hg compared with 6.4–7.6 mm Hg in the timolol group. Retention rates improved compared with earlier reports but remained relatively low in the mid-term (91% at day 60 and 48% at day 90). However, this may underestimate retention due to the lack of ability of transillumination to locate small remnants of OTX-TP by visualization [22]. Finally, in a multicenter randomized phase 3 trial comparing OTX-TP to a placebo vehicle insert, OTX-TP showed generally good tolerability, with no serious ocular adverse events observed. The most common ocular adverse events (AEs) included dacryocanaliculitis (approximately 7% in OTX-TP vs. 3% in placebo) and lacrimal structure disorder (around 6% in OTX-TP vs. 4% in placebo). The tolerability of the implant improved over time. However, the trial did not prove meaningful preponderance in mean IOP reduction, likely due to its sample size and short duration. According to personal communication with the manufacturer, the OTX-TP system is no longer being built up as it did not show superiority to eye drops [23]. In 2010, Kompella et al. and subsequently Goldberg et al. in 2011 investigated the Latanoprost punctal plug delivery system (PPDS) developed by QLT (Vancouver, BC, Canada). Later, the L-PPDS (Latanoprost Punctal Plug Delivery System) was acquired by Mati Therapeutics Inc. (Austin, TX, USA) They employed an L-shaped plug containing a nonbiodegradable Latanoprost core, insertable at the slit lamp, with two plugs placed into one eye. Each plug contained 70.5 mg of Latanoprost, exhibiting slow drug release over 30 days. After four weeks of L-PPDS treatment, a statistically significant mean reduction in IOP from baseline (−5.7 mm Hg) was observed. The L-PPDS demonstrated good tolerability throughout the study period, with AEs comparable to those reported for commercial punctal plugs. This extracellular and minimally invasive drug delivery system demonstrated clinically significant and sustained (4 weeks) reduction in IOP [17,24]. Although punctal plugs are believed to be relatively safe, their use is commonly associated with epiphora, conjunctival irritation, punctal plug extrusion, and, less commonly, with inflammatory conditions such as dacryocystitis. There can also be a slight irritation or itchiness in the tear duct area after the first insertion, which usually disappears after acclimatization. Furthermore, an external plug can be forced down into the canaliculus through insertion or lost afterwards. The same problem can occur with intracanalicular plugs [25,26]. The inconstant delivery from the punctum to the tear film and for the low drug doses that punctal plugs, as drug carriers, can handle may also limit the IOP-lowering effect.

## 3. Conjunctival Fornix Delivery System

Conjunctival fornix delivery systems include implants, gels, or particles designed for prolonged drug release (Table 2).

The implant system comprises a non-toxic sterile device shaped to fit the conjunctival cul-de-sac. Like other delivery systems described in this review, it is developed to administer glaucoma therapy more efficiently, avoiding the patient’s compliance problem. Many recent studies focus on the search for a new method to extend the drug permanence on the ocular surface, considering that the half-life of eyedrops is about 4 min after administration [35]. These devices can be distinguished as soluble, insoluble, and bioerodible. The drug release occurs by diffusion for the non-degradable inserts and by dissolution or erosion for the degradable ones. Their main advantages include precise dosage, increased bioavailability, lack of preservatives, continuous effectiveness without peaks for a lasting period ranging from hours to days, and reduced number of administrations. Unfortunately, some disadvantages were reported, such as foreign body sensation, eye discharge, occasional device loss, and difficulty placing and removing the insert.

The Pilocarpine Ocusert insert (developed by Alza corporation) [27], approved for clinical use in 1975, was a polymer-membrane system composed of two elliptical membranes 13.4 mm long and 13.7 mm wide, among which is positioned the pilocarpine core. Designed to be worn in the upper or lower conjunctival fornix, it supplied the drug for seven days through diffusion with two different rates: 20 µg per hour or 40 µg per hour. This system avoided the pulsatile fluctuations of the eye drops by maintaining a constant rate of the drug on the eye’s surface. Many adverse events peculiar to pilocarpine drops, such as brow ache and blurred vision caused by myopic shift, were reduced with Ocusert. However, many side effects were reported, such as dislodgement, foreign body perception, burning sensation, conjunctival hyperemia, and high cost [36]. This device was used until 1993, when it was withdrawn from the market.

The Topical Bimatoprost Ocular Insert [28] is a preservative-free ocular ring with an inner polypropylene structure surrounded by a silicone matrix containing 13 mg of Bimatoprost. The diameter of this ring varies between 24 and 29 mm depending on the intercanthal distance. Drug delivery is based on passive diffusion of the drug in the tear film with a declining dose from day 0 (35 µg/day) to day 180 (6 µg/day). A multicenter, phase 2, randomized, prospective study assessed the efficacy and safety of a Bimatoprost insert compared to a 0.5% timolol ophthalmic solution administered twice a day in 130 patients with OAG and OHT over a six-month period. Ocular and non-ocular adverse events were more prevalent in the Bimatoprost insert group. The most frequent were eye discharge, conjunctival hyperemia, punctate keratitis, eye itching, and ocular discomfort. However, all the ocular AEs were mild to moderate, and patients fully recovered without consequences. A clinically relevant IOP reduction of −3.2 to −6.4 mm Hg was reached (more than 20% compared with the baseline) with the Bimatoprost insert compared with a decrease of −4.2 to −6.4 mm Hg for the timolol group. Although a considerable IOP reduction was reached, non-inferiority of the Bimatoprost insert was not demonstrated: non-inferiority was set as a mean difference lower than 1.5 mm Hg, and this goal was not reached in the Bimatoprost group. The authors attribute this result to the small sample size and the probable paradoxical response of prostaglandins, which are less effective when frequently administered. At the end of this study, 75 patients joined an open-label single-arm study for 13 months [29]. A median reduction of 4 mm Hg was observed, proving that the Bimatoprost insert can lead to a clinically relevant decrease in IOP over 19 months.

A combination ring containing Bimatoprost and timolol has been studied in a clinical trial. However, results are still unavailable (nct02742649).

Timolol maleate-loaded inserts designed to fit in the conjunctival cul-de-sac were investigated by Nair et al. [30] These devices are composed of a polymeric matrix with a solvent evaporation complex incorporating both a hydrophilic polymer (sodium alginate) and a hydrophobic polymer (ethyl cellulose), with polyethylene glycol (PEG) acting as a plasticizer. Different formulations (F1–F7) were developed by changing the concentrations of the polymer, which was found to influence drug release negatively. The formulation with the best pharmacologic properties (F4) was selected for use in vitro and then in vivo. In vivo studies on rabbits compared F4 formulation and marked timolol maleate drop solution. A faster response and longer persistence of the drug were observed in the former group.

Recent advancements in pharmaceutical research have explored the utilization of liposomes as a novel formulation to prolong the effects of eye drops and enhance their efficacy. In 2022, Bigdeli et al. found the optimized liposomal formulation containing timolol maleate (TM) and brimonidine tartrate (BT) and studied its effectiveness in 36 rabbits [34]. The IOP was successfully increased in the right eye of these rabbits by injecting Hydroxypropyl methylcellulose (HPMC) polymer into the anterior chamber. Then, the rabbits were divided into six groups: the control group, the ones that received drug-loaded liposomes (TM/BT, only TM, only BT) or a drug-free liposomal formulation, and finally, the one in which aqueous solutions of TM/BT were instilled. The IOP was measured before HPMC polymer injection in the AC, 24 h after, and 2 h after each eye drop instillation (27, 41, 51, 65, 75, 89, 99, 113, 123, and 137 h after starting the injection in the right eye AC). In each group, the study ended when eye pressure returned to its baseline value. The results showed that 27 h after the injection, both formulations were capable of substantially lowering the IOP compared with the control group (*p* < 0.001), and specifically, the liposomal formulation was more effective in lowering the IOP compared with the simple formulation of the aqueous solution. Specifically, the selected liposomal formulation made by thin layer hydration increased the efficacy of the timolol maleate and brimonidine tartrate combination could provide sustained release for 12 h.

Nanotechnology has led to the development of innovative systems for the administration of hypotensive drugs such as timolol. A study by Burcu Uner et al. [37] investigated timolol-loaded ethosomes, which are lipid-based vesicles characterized by their small size and elasticity. These ethosomes enhance drug permeation, and both in vitro and in vivo tests showed no significant difference (*p* > 0.05) between a single application and three applications of the conventional drug, suggesting potential benefits for glaucoma treatment.

Another study [38] examined the effectiveness of timolol encapsulated in nanogels made from N-isopropylacrylamide (NIPA) and acrylic acid (AAc). This in vivo study in animal models showed that a single application could reduce and stabilize intraocular pressure (IOP) for 48 h. Additionally, nanogels containing timolol maleate and biopolymers such as chitosan and alginate demonstrated sustained drug release and improved permeation, indicating a promising strategy for glaucoma therapy [39].

Furthermore, a nanofibrous drug delivery system (DDS) was developed as an alternative to eye drops for brinzolamide (BRZ) delivery [40]. This system utilized β-cyclodextrin (β-CD), hydroxypropyl cellulose (HPC), and polycaprolactone (PCL) and exhibited sustained drug permeation through sheep corneas, achieving therapeutic levels. This nanofibrous DDS offers a significant advantage over traditional eye drops by providing extended drug delivery primarily through the cornea.

The New Ophthalmic Delivery System (NODS) in a novel device where the drug is loaded into a water-soluble polyvinylalcohol (PVA) film, which dissolves when in contact with the tears in the lower conjunctival sac, detaching from the other parts of the device.

Kelly et al. [31] demonstrated that pilocarpine delivered in NODS is well-tolerated, and its dosage is eight times smaller compared with conventional eye drops with a consequent reduced systemic absorption.

Greaves et al. [32] utilized radiolabeled NODS containing pilocarpine nitrate for a scintigraphic study to evaluate precorneal residence and pharmacodynamics in human subjects. This study showed eight-fold precorneal bioavailability of the pilocarpine NODS compared with a 25 µL drop of 2% pilocarpine nitrate solution.

Later, Diestelhorst et al. [33] analyzed the tolerability of NODS, comparing it with the tolerability of Isopto-Naturale R eye drops. The study underlined that NODS were less tolerated than conventional drops through questionnaires. However, this problem was related not to the device itself but to the mechanism of delivery. In many cases, the medicated plaque did not detach from the rest of the delivery system.

Finally, numerous natural polymers have been integrated into drug delivery systems to produce drops that can persist longer on the eye’s surface. An example of these polymers is collagen, which has been utilized extensively in ocular applications, including collagen shields. These devices have been loaded with different drugs like steroids or antibiotics. However, their use decreased due to limitations such as non-transparency and toxicity of the cross-linking agents.

Agban et al. [41] devised cross-linked collagen shields employing metal oxide nanoparticles (NPs) such as titanium dioxide (TiO_2_), zinc oxide (ZnO), and zinc oxide encapsulated with polyvinylpyrrolidone (ZnO/PVP) as cross-linking agents for the controlled release of pilocarpine hydrochloride. The results of this study underlined a release of pilocarpine hydrochloride in vitro over 14 days.

## 4. Contact Lens for Ophthalmic Drug Delivery

Contact lenses serve a multifaceted role in ophthalmic care, being utilized not only for the correction of visual impairments but also for protecting compromised corneal surfaces. Ongoing investigations have unveiled their potential as an alternative method for the delivery of ocular medications (Table 3).

In 1960, Wichterle [51] drew attention to drug-laden contact lenses, and the interest in this area has grown [8]. Drug-laden contact lenses are thin, curved lenses applied to the corneal surface. They can be made of different materials, but silicone hydrogel lenses are currently the best for drug carriage, having good oxygen permeability and being safely worn for 30 consecutive days [52]. When the drug is released from the lens, it maintains high concentrations for approximately 30 min into the tear film, compared with 4 min on average for eye drops [44,47]. For this reason, drug-laden contact lenses seem to be an excellent alternative to eye drops for managing anterior segment disease.

In 1965, Sedlacek pioneered the development of a contact lens soaked in a 1% homatropine aqueous solution to achieve prolonged mydriasis in patients [53]. Shortly thereafter, North devised a lens immersed in a 4% pilocarpine solution with the aim of reducing IOP [54]. Soaking is the first, simplest, and cheapest method of drug-loading hydrogel contact lenses. However, the loading capacity of the drug is limited, and the duration of its effect is short (about 24 h). In 2008, Kim and colleagues designed hydrogel soft contact lenses for extended wear, in which, although the drug-loading method is soaking, the particular material allows the drug (timolol) to be carried for a more extended period, ranging from weeks to months [43]. Moreover, Peng in 2010 demonstrated that silicone hydrogel lenses medicated with vitamin E (10–40%) significantly prolonged the release duration of hydrophilic drugs (e.g., timolol) while preserving lens transparency (vitamin E has a shorter wavelength than visible light) [32].

Subsequently, other authors have developed hydrogel contact lenses with embedded ionic components that interact with the drugs in which the lenses are soaked [55].

In 1998, a device incorporating two contact lenses fused with a central cavity for drug insertion was developed by Nakada and Sugiyama [56]. Following this, the concept was further refined by other researchers, who introduced the term “molecular imprinting” to describe the process of creating cavities within contact lenses with a high affinity for the drug [57]. This method garnered interest due to its simplicity of refilling: by immersing the lens in the drug solution, the drug is drawn into the contact lens, resulting in the formation of a polymer—a compound composed of numerous smaller molecules [44]. However, it is essential that the composition of the lens is tailored specifically to the drug when utilizing this technique. In 2002, Hiratani et al. demonstrated that contact lenses loaded with timolol, prepared using molecular imprinting, could sustain drug release for over 24 h in a saline solution [42].

Another promising approach is the “hypercritical fluid method”, classically applied for bulk transport and extraction, which consists of separating one component from another using supercritical fluids as the extracting solvent. The drug is dispersed in the supercritical solvent and interacts with the contact lens. This method is effective for both hydrophilic and hydrophobic drugs and can be combined with molecular imprinting, allowing a more significant drug loading and extended release [45,58].

Another strategy is the encapsulation of drugs in colloidal nanoparticles, allowing more extended drug durability at the level of the cornea [59]. However, these nanoparticles form aggregates and reduce lens transparency and oxygen permeability [60]. Jung and Chauhan in 2012 published a study on the dispersion of timolol encapsulated in cross-linked polymeric nanoparticles within contact lenses, which extended the length of drug release to 2–4 weeks [46]. Liposomes are nanoparticles that can be embedded in the lens matrix or at the level of the lens surface, and these have been shown to increase the duration of drug release, enhance the stability of the tear film, and increase the thickness of the lipid layer [61]. In addition, microemulsions are promising nanoparticles. They have the advantage of easy preparation and sterilization. However, their use is still challenging because their stability is poor (for example, timolol dissolved in ethyl butyrate releases very rapidly) [60].

Finally, Ciolino et al. have developed contact lenses with a drug-polymer film. In this case, by altering either the type or molecular weight of the polymer, drug release characteristics can be modified, showing promising potential [50].

Unfortunately, the existing literature on drug-eluting contact lenses remains predominantly preclinical, with studies primarily conducted in animal models. Nonetheless, we draw attention to two noteworthy investigations that have underscored the safety and efficacy of such devices. In 2012, Jung pioneered the development of extended-wear contact lenses infused with dispersed nanoparticles of PGT (propoxylated glycerol triacrylate) loaded with timolol. This loading was achieved by immersing the lens in an ethanol solution containing the particles. Both in vitro and in vivo experiments conducted on Beagle dogs exhibited a consistent reduction in IOP [48]. Similarly, in 2015, Hsu demonstrated in laboratory and animal models that contact lenses loaded with Dorzolamide and timolol, when co-loaded with vitamin E, could sustain prolonged drug release for approximately two days. This extended release led to a further reduction in IOP compared with traditional Cosopt eye drops [49].

The advantages associated with contact lenses suggest its potential for broader acceptance in the future. They offer ease of use, maintain drug concentrations effectively over extended periods, reduce administration frequency compared with eye drops, and can simultaneously correct visual defects. However, contact lenses pose certain risks, including the potential for sight-threatening microbial keratitis. Challenges related to biocompatibility, patient comfort, optical clarity, drug loading, and delivery persist [62]. Moreover, medications used for glaucoma treatment necessitate continuous wear of contact lenses, even overnight, which may compromise oxygen permeability, leading to corneal edema [62]. Additionally, in the elderly population, where glaucoma prevalence is high, both insertion and wearing of contact lenses can be challenging due to reduced and poor-quality tear film, often causing discomfort [63]. Furthermore, prolonged use of drug-eluting contact lenses can result in systemic absorption of medications through the conjunctiva, posing risks, especially for patients with conditions such as asthma or heart disease [64]. Striking a balance between effective drug release, intraocular pressure reduction, and maintaining transparency and adequate oxygen permeability in contact lenses remains a challenge. Despite ongoing research, there is currently no FDA-approved device for these reasons. However, research on drug-eluting contact lenses persists due to their user-friendly nature and direct contact with the corneal surface, offering promising avenues for future development [8].

## 5. Periocular Drug Delivery Systems

The main periocular routes of drug delivery include subconjunctival, sub-Tenon, and posterior juxtascleral pathways.

However, the most recent studies in the field of glaucoma predominantly focused on the subconjunctival route due to its minimally invasive nature and proximity to the target tissues (Table 4) [65].

The subconjunctival route enables, in fact, continuous drug delivery to the anterior segment tissues by positioning the drug in close proximity to the limbus, cornea, and ciliary body. Advantages of this route include the wide range of delivery systems available, such as implants, microspheres, nanospheres, liposomes, and gels, as well as the ability of particles to remain in place for extended periods, potentially facilitating sustained drug delivery for several months [69,70]. Larger particles have been found to be more suitable than micro- and nano-particles, with a more hydrophobic surface enhancing the retention of negatively charged nanoparticles [70]. However, these drug delivery systems may be visible and could induce visible hemorrhage on the eye surface. Most trials focusing on this route of administration have explored the use of liposomes.

Wong et al. proved that liposomal Latanoprost injected subconjunctivally in rabbits was more effective than daily eye drops in reducing IOP for approximately 50 days with good tolerability in all the subjects treated [66]. In 2012, the same group developed Latanoprost-loaded egg-phosphatidylcholine (EggPC) liposomes and evaluated their stability, tolerability, and efficacy in vivo using 16 rabbits divided into two groups: one group received injections of Latanoprost-loaded EggPC liposomes, while the other received Latanoprost eye drops. Results indicated that a single injection of these liposomes was not only effective in lowering IOP for up to 90 days but also achieved a greater reduction compared with daily topical administration of Latanoprost (4.8 ± 1.5 vs. 2.5 ± 0.9 mm Hg; *p* < 0.001). No signs of inflammation were observed in the eyes during slit-lamp examination [67]. However, it is noteworthy that these findings were obtained in rabbits, in which the prostaglandin analogs induce a significantly lower IOP reduction compared with humans due to the different sensitivity and localization of the receptors involved [71]. In 2014, the research team initiated an open-label safety and efficacy study involving six humans affected by ocular hypertension or primary open-angle glaucoma, focusing on both short-term and long-term (3 months) efficacy of a single subconjunctival injection of nanoliposomes (100 nm) containing Latanoprost [68]. One hour after the injection, intraocular pressure decreased from 27.55 ± 3.25 mm Hg to 14.52 ± 3.31 mm Hg (range 10–18 mm Hg), with an intraocular pressure reduction ranging from 37% to 63% and a mean decrease of 13.03 ± 2.88 mm Hg (range 9–17 mm Hg), which was statistically significant at each post-treatment visit (*p* = 0.001 to 0.049). All six subjects experienced an immediate IOP reduction of 9 mm Hg or more (≥20%). After three months, five out of six subjects showed lower IOP compared with baseline, especially those who had been using Latanoprost drops before enrollment; the one patient whose IOP after three months returned to baseline had previously been treated with timolol eye drops. Regarding safety, all patients tolerated the injection well, reporting only mild ocular discomfort immediately after the injection. The subconjunctival liposome delivery system is still not approved to date, and the long-term potential adverse effects of nanomedicine are still unknown.

In 2017, a multicenter 12-week phase 1/2a clinical trial was conducted to assess the effectiveness and tolerance of the Eye-D VD-101 insert, a subconjunctival implant built to release Latanoprost over a 12-month period. The study enrolled 77 patients and compared three different doses of the VS-101 insert with Latanoprost eye drops applied once daily. Results at the 12-week mark showed a 24% decrease in IOP from the baseline, accompanied by mostly mild adverse events and favorable tolerability [9].

Graybug Vision demonstrated that the subconjunctival injection of GB-401, an encapsulated microparticle formulation of a beta-adrenergic antagonist prodrug that was able to lower IOP by hydrolyzing into an active agent. The IOP reduction was significant within the first week after the injection and was sustained with maximum IOP lowering of ~20% for over two months. These results were obtained in pigmented rabbits both in vitro and in vivo, but human glaucomatous patients are being recruited for the upcoming first-in-human phase 1/2a study [9].

Although the results of periocular drug delivery systems studies are promising, their main limitation is that they were conducted mostly on rabbits; more studies on human patients are necessary to better define the efficacy and tolerability of these drugs.

## 6. Intracameral Delivery System

The corneal and precorneal barriers represent significant impediments to drug penetration into the eye, thereby diminishing intraocular bioavailability. The intracameral approach, involving direct injection of therapeutic agents into the anterior chamber, offers a solution to these challenges posed by extraocular routes (Table 5). This approach yields higher concentrations of drugs in the aqueous humor and adjacent ocular tissues, enhancing therapeutic efficacy while requiring smaller doses of the drug [72].

Currently, two sustained-release prostaglandin implants have received the U.S. Food and Drug Administration (FDA) approval, with several others undergoing development.

In March 2020, the U.S. FDA granted approval for “Durysta”, marking the introduction of the first intracameral biodegradable implant containing Bimatoprost (BimSR), designed for single-use applications. This implant takes the form of a solid, rod-shaped structure comprising 10 µg of Bimatoprost encapsulated within a poly(D, L-lactide-co-glycolide) polymer matrix. Post-implantation, the matrix gradually degrades, facilitating the sustained, non-pulsatile release of the drug through zero-order kinetics [72]. Pharmacokinetic analysis revealed that 80.5% of the Bimatoprost payload was released by day 51, reaching 99.8% by day 80, with no detectable drug levels beyond 4.5 months post-injection. The intracameral administration route enables direct delivery of the drug to the iris and ciliary body, thereby reducing the overall medication requirement for achieving therapeutic effects. The 10 µg of drug released from the implant is equivalent to the dosage contained in a single drop of Bimatoprost 0.03% ophthalmic solution. Additionally, minimal to undetectable drug levels were observed in non-target extraocular tissues, mitigating the risk of adverse events such as orbital fat atrophy [34]. Prostaglandin analogs (PGAs) are commonly employed as first-line treatments due to their established efficacy and safety profile. While PGAs primarily lower IOP by enhancing aqueous humor outflow via the uveoscleral pathway, intracameral BimSR may offer additional mechanisms for IOP reduction. Pharmacokinetic investigations revealed that following BimSR injection, drug concentrations were 4400-fold higher compared with once-daily topical Bimatoprost 0.03% eye drops, with intact Bimatoprost being the predominant drug moiety detected in the iris-ciliary body, as opposed to Bimatoprost acid observed with topical eye drops. Furthermore, supplementary IOP reduction was demonstrated when BimSR was administered to normotensive cynomolgus monkeys already receiving topical Bimatoprost eye drops. Hence, it is plausible that the Bimatoprost implant provides IOP reduction through alternative mechanisms beyond those offered by Bimatoprost eye drops [76]. The implantation procedure necessitates the use of an aseptic technique conducted under an operating microscope. A 28-gauge needle is utilized to insert the implant into the anterior chamber, typically through the temporal or superotemporal clear cornea. Following the injection, it is recommended that the patient maintain an upright position for approximately one hour to facilitate the settling of the implant in the inferior angle [77]. A 24-month, paired-eye, phase 1/2 clinical trial was undertaken to evaluate the safety and effectiveness of the Bimatoprost implant in individuals with OAG. Participants were divided into four groups, each receiving different dosages of the drug (6, 10, 15, and 20 μg). An interim analysis published at the six-month mark revealed that each dosage of BimSR resulted in a comparable reduction in IOP compared to once-daily Bimatoprost 0.03% eye drops. Additionally, it was observed that the reduction in IOP was dose-dependent for at least 12 weeks, and subsequent administrations were equally effective as the initial one. Following this, the approval of Durysta was based on the findings from two identical 20-month phase 3 clinical trials, known as ARTEMIS 1 and ARTEMIS 2. These trials were characterized by their prospective, multicenter, randomized design and compared the effects of a 10 or 15 μg Bimatoprost implant with twice-daily topical timolol maleate 0.5% in patients with open-angle glaucoma or ocular hypertension. Primary endpoints included IOP and IOP change from baseline through week 12. Safety assessments encompassed various measures such as best-corrected visual acuity (BCVA), biomicroscopy examination, gonioscopy, fundus examinations, corneal endothelial cell density (CECD) evaluation, corneal thickness evaluation, and treatment of emergent adverse events (TEAEs). Particular attention was paid to corneal AEs (mainly corneal endothelial cell loss and/or corneal edema) and inflammatory adverse events (especially keratitis, iritis, and vitritis). Results from ARTEMIS 1 indicated that both 10 μg and 15 μg Bimatoprost implants were non-inferior to timolol in terms of IOP and IOP changes from baseline. Furthermore, subsequent administrations of the implants demonstrated sustained efficacy, with many patients requiring no additional IOP-lowering treatment, even during extended safety follow-ups. However, the incidence of TEAEs, including corneal changes and inflammatory reactions, was higher in the Bimatoprost implant groups compared with the timolol group, particularly with repeated dosing. The most frequent side effects were conjunctival hyperemia, eye irritation, foreign body sensation, and eye pain (some probably caused by the injection procedure). There were no reports of eyelash growth or periorbital fat atrophy [78]. Similarly, ARTEMIS 2 showed favorable outcomes for the 10 μg and 15 μg Bimatoprost implant groups compared with the timolol group in terms of mean IOP and mean change from baseline IOP. The efficacy of repeated administrations was also confirmed. Despite the promising efficacy, concerns were raised regarding adverse events, particularly corneal changes such as corneal endothelial cell loss, which appeared to be higher than expected, especially with repeated administrations and in the 15 μg implant group [78]. Another noteworthy finding from ARTEMIS 1 and 2 is the reduced necessity for additional IOP-lowering treatments among many patients treated with BimSR, persisting even after four months from the last injection. Stramer et al. have reported that BimSR injection may sustain outflow by inducing a significant upregulation in MMP1 and downregulation of fibronectin. Notably, the dramatic upregulation in MMP1 and downregulation of fibronectin are observed exclusively with Bimatoprost at high concentrations and not with BFA [76]. In conclusion, the risk/benefit evaluation favored the 10 μg Bimatoprost implant over the 15 μg Bimatoprost implant. However, many other BimSR phase 3 trials are underway or pending the release of results.

The iDose, developed by Glaukos Corporation based in San Clemente, California, United States, is a sustained-release intraocular implant containing a unique formulation of travoprost. This implant consists of three primary components: (1) a scleral anchor for insertion and secure attachment to the inner scleral wall via the trabecular meshwork; (2) a titanium body serving as a drug reservoir; and (3) a membrane designed to release travoprost gradually over a targeted duration ranging from six to twelve months. Insertion and removal of the implant are performed under an operating microscope. A phase 2 trial, conducted as a multicenter, randomized, double-masked study, compared the efficacy of iDose with two different rates of drug elution to twice daily topical timolol 0.5%. The trial enrolled 154 patients, with 51 receiving the fast-release formulation of iDose, 54 receiving the slow-release formulation, and 49 receiving timolol 0.5% eye drops. Results showed comparable mean IOP reductions at the end of the first 24 months among the groups. In addition to its efficacy, the iDose device demonstrated a favorable safety profile during phase 2 clinical trials, with no significant corneal or conjunctival adverse events reported. The FDA granted approval to iDose^®^ TR in December 2023 for managing IOP in patients with open-angle glaucoma or ocular hypertension. However, it is contraindicated in individuals with active or suspected ocular infections, corneal endothelial cell dystrophy, prior corneal transplants, or hypersensitivity to its components. Caution is advised for patients with certain angle abnormalities. FDA approval was based on two phase 3 pivotal trials (GC-010 and GC-012) involving 1150 subjects across 89 clinical sites. Both trials successfully met primary efficacy endpoints over a 3-month period and demonstrated favorable safety profiles over 12 months. While iDose TR showed non-inferiority to timolol ophthalmic solution in reducing IOP during the initial 3 months, it did not maintain this non-inferiority over the subsequent 9 months. At the 12-month follow-up, a significant portion of iDose TR subjects were medication-free, with high tolerability and subject retention rates. Ocular adverse reactions were mostly mild and transient, including increases in IOP, iritis, dry eye, and visual field defects [79].

ENV515, developed by Envisia Therapeutics located in Morrisville, NC, USA, is a biodegradable intracameral implant containing travoprost. This implant, shaped like a rod, is produced using innovative particle replication in non-wetting templates technology, allowing for the creation of sterile nanoparticles with an extended-release formulation of travoprost. Injection of the implant into the anterior chamber is performed using a specialized injector under an operating microscope, typically resting stably in the inferior iridocorneal angle. Prior to human clinical trials, preclinical studies were conducted in normotensive and hypertensive beagles, varying in implant size, drug concentration, and number. These studies showed a reduction in intraocular pressure following implantation, with the device being well-tolerated and causing minimal adverse effects, primarily transient ocular irritation related to the surgical procedure. In a phase 2a clinical trial involving 21 patients with open-angle glaucoma, ENV515 was compared to once-daily topical travoprost and met the primary endpoint of non-inferiority by day 25. Another clinical trial evaluated ENV515 against topical timolol 0.5% eye drops in open-angle glaucoma patients, demonstrating an IOP reduction of 6.7 ± 3.7 mm Hg and confirming non-inferiority to timolol over 11 months [69,74,78].

## 7. Conclusions

The primary goal of managing glaucoma patients is to enhance their well-being and quality of life within a sustainable healthcare framework. Visual functionality plays a pivotal role in determining quality of life, with individuals experiencing minor visual impairment generally maintaining an acceptable standard of living. However, as visual impairment worsens, overall well-being declines. Additionally, factors such as treatment costs and drug side effects significantly impact patients’ quality of life, including the inconvenience faced during chronic therapy. The sustained drug delivery systems (SDDSs) explored in this review can have a significant impact on glaucoma management and patients’ quality of life. The advantages are substantial: by releasing medication gradually over an extended period, SDDSs can significantly reduce the frequency of dosing, thereby enhancing patient adherence and slowing disease progression. Improved adherence through SDDSs is critical for maintaining optimal IOP control, essential for preventing optic nerve damage and vision loss. SDDSs can be precisely engineered for targeted drug delivery to specific ocular tissues, such as the anterior chamber or ciliary body. This targeted delivery approach increases drug efficacy by concentrating medication at the site of action while reducing systemic exposure, thus minimizing side effects. This is particularly advantageous for patients at elevated risk of systemic side effects or those taking multiple medications. Moreover, SDDSs alleviate the burden of frequent daily dosing, simplifying the treatment regimen, and this could improve patient convenience and quality of life. Research indicates that traditional eye drop regimens often cause frustration and reduce the quality of life for glaucoma patients. By making the treatment regimen less demanding, SDDS can empower patients to manage their condition more effectively and enhance their overall well-being. However the long-term impacts of SDDS in glaucoma treatment need further exploration. Most clinical trials on SDDSs focus on short-term efficacy and safety. To fully understand potential side effects, drug interactions, and the overall effect on glaucoma progression over extended periods, long-term studies are essential. Monitoring potential issues like inflammation, irritation, or scarring is crucial in these studies. Ensuring a consistent and controlled release of medication throughout the intended duration is critical. Long-term research can uncover potential problems, such as drug degradation within the SDDS or changes in release rates over time. Moreover, a comprehensive analysis is required to balance the initial costs of SDDSs against potential long-term savings. This includes factors like improved adherence, which reduces medication waste, fewer complications leading to less need for additional treatments, and reduced office visits due to less frequent dosing. Finally, it is very important to consider the central role of the patient, who should receive clear and thorough information about the benefits, proper usage, and possible limitations of SDDSs. Providing a variety of SDDS formulations and delivery methods can cater to individual patient needs and preferences [80].

## Figures and Tables

**Table 1:** Punctal plugs delivery systems.

Authors	Type of Study	Year	Device	Drugs	Results
Chen et al. [14]	Case-control study	2020	Temporary Collagen Punctal Plugs		A statistically significant IOP diminution in the case group compared with the control group. DED ameliorated significantly in the case group rather than in the control group.
Sherwin et al. [15]	Randomized controlled trial	2018	Punctal plugs in patients using prostaglandin analog monotherapy		Punctal plugs resulted in a significantly lowered IOP (MD 1.5 mm Hg, 95% CI 0.1–2.9, *p* = 0.032).
Perera et al. [16]	Initial feasibility, prospective, single-arm	2016	OTX-TP	Travoprost	OTX-TP can reduce IOP by 24% (day 10) and 15.6% (day 30).
Kompella et al. [17]	Phase 2 trial	2010	L-PPDS	Latanoprost	A mean reduction in IOP by 3.5 mm Hg at the end of 4 weeks, with 36% of patients showing a decrease in IOP of more than 5 mm Hg.

**Table 2:** Conjunctival fornix delivery system.

Authors	Type of Study	Year	Device	Drugs	Results
Alza Corporation [27]	Phase 3 trial, randomized, multicenter, masked (previously on the market)	1975	Pilocarpine Ocusert	Conjunctival fornix delivery system, pilocarpine	Positive outcomes in IOP lowering but also many side effects. Withdrawn from the market in 1993.
James D. Brandt et al. [28]	Double-masked, randomized, multicenter, phase 2 study	2016	Topical Bimatoprost Ocular Insert	Conjunctival fornix delivery system, Bimatoprost	Bimatoprost Ocular Ring did not reach the non-inferiority standard in lowering IOP compared with timolol eye drops.
James D. Brandt et al. [29]	A 13-month open-label extension (OLE) study	2017	Topical Bimatoprost Ocular Insert	Conjunctival fornix delivery system, Bimatoprost	A median reduction of 4 mm Hg was reached over a follow-up period of 19 months.
Nair et al. [30]	Preclinical study	2018	Timolol maleate-loaded inserts	Conjunctival fornix delivery system, timolol	Faster response and longer persistence of the drug in timolol maleate-loaded inserts compared with timolol maleate drop solution.
Kelly et al. [31]	Single-dose crossover study	1989	New Ophthalmic Delivery System (NODS)	Conjunctival fornix delivery system, pilocarpine	The bioavailability of NODS is eight times greater compared with conventional eye drops.
Greaves et al. [32]	Gamma scintigraphic study on 12 volunteers	1992	Radiolabeled New Ophthalmic Delivery System (NODS) loaded with pilocarpine nitrate	Conjunctival fornix delivery system, pilocarpine	IOP-lowering; more significant pupil diameter reduction and higher bioavailability compared with a 25 µL drop of 2% pilocarpine nitrate solution.
M. Diestelhorst [33]	Open-label, crossover study	1994	Radiolabeled New Ophthalmic Delivery System (NODS)	Conjunctival fornix delivery system	NODS were less tolerated than conventional eye drops.
Bigdeli et al. [34]	In vivo (rabbits)	2023		Timolol Maleate-Brimonidine tartrate-loaded liposomes	Both liposomal and aqueous formulations reduce IOP, although liposomal formulation is more efficient (rabbits).

**Table 3:** Drug-laden contact lenses.

Authors	Type of Study	Year	Device	Drugs	Results
Hiratami and Alvarez-Lorenzo [42]	Preclinical trial	2002	Soft contact lenses of a cross-linked hydrogel loaded with molecular imprinting method	Timolol	This method improves the drug loading capacity and prolongs timolol release for more than 24 h
Kim et al. [43]	Preclinical trial	2007	Soaked silicone hydrogel soft contact lenses	Timolol	This method increases drug release for about 15–20 days
Peng et al. [44]	Preclinical trial (animal model)	2012	Silicone hydrogel contact lenses loaded with vitamin E	Timolol	This method increases the bioavailability of the drug and reduces systemic drug uptake
Braga et al. [45]	Preclinical trial	2011	Drug loaded into commercial soft contact lenses with a supercritical solvent impregnation process	Acetazolamide	With this method, it is possible to control acetazolamide-loaded amounts and adjust the drug release levels
Jung and Chauhan [46]	Preclinical trial	2012	Drug encapsulating highly cross-linked nanoparticles in contact lenses	Timolol	This method increases the duration of drug release to 2–4 weeks
Peng et al. [47]	Preclinical trial (animal model)	2012	Commercial soft contact lenses incorporating vitamin E	Timolol	This method increases the drug release duration and lowers the IOP by continuously wearing
Jung et al. [48]	Preclinical trial (animal model)	2013	Nanoparticle-loaded contact lenses	Timolol	This method allows a constant reduction in IOP
Hsu et al. [49]	Preclinical trial (animal model)	2015	Vitamin E-loaded contact lenses	Timolol and Dorzolamide	This method ensures prolonged release of the drug and further reduces IOP
Ciolino et al. [50]	Preclinical trial (animal model)	2016	Contact lenses with a drug-polymer film	Latanoprost	This method is effective as delivery with daily Latanoprost ophthalmic solution

**Table 4:** Periocular drug delivery systems.

Authors	Type of Study	Year	Device	Drugs	Results
Wong et al. [66]	In vivo (rabbits)	2014		Subconjunctival Latanoprost injection	More significant IOP reduction than Latanoprost eye drops for about 80 days (2 injections-day one and day 50) (rabbits).
Wong et al. [67]	In vivo (rabbits)	2012		Latanoprost-loaded EggPC liposomes	A single injection of these liposomes lowers the IOP for up to 90 days, a more significant IOP reduction than Latanoprost eye drops (rabbits).
Wong et al. [68]	Open-label, non-comparative study	2014		Latanoprost-loaded nanoliposome	A single injection decreased IOP immediately in all six subjects; after three months, IOP was lower than baseline in all subjects except one (humans).
Kesav et al. [9]	Phase 1/2a clinical trial, multicenter	2017	Eye-D VD-101 insert	Latanoprost-releasing subconjunctival insert	The implant reduces IOP by 24% from baseline at 12 weeks. Non-inferior to Latanoprost eye drops (humans).
Kesav et al. [9]	In vivo (rabbits)	2018	GB-401 injection	Beta adrenergic prodrug subconjunctival injection	The implant reduces IOP within the first week and up to 20% from baseline at 2 months (rabbits).

**Table 5:** Intracameral delivery systems.

Authors	Type of Study	Year	Device	Drugs	Results
J. Bacharach et al. [73]	Phase 3 trial, randomized, multicenter, masked	2021	Durysta	Intracameral sustained released Bimatoprost	BimSR 10 μg and 15 μg were non-inferior in lowering IOP to timolol eye drops.
F.A. Medeiros et al. [74]	Phase 3 trial, randomized, multicenter, masked	2020	Durysta	Intracameral sustained released Bimatoprost	BimSR 10 μg and 15 μg were non-inferior in lowering IOP to timolol eye drops.
John P. Berdahl [75]	Phase 2 trial, randomized, multicenter, masked	2023	Idose	Intracameral sustained released travoprost	The fast-release and slow-release formulations were non-inferior in lowering IOP to timolol eye drops.
Courtesy of the producer	Phase 2 trial, randomized, multicenter, masked		ENV515	Intracameral sustained released travoprost	ENV515 was non-inferior to topical travoprost on day 25 and to topical timolol 0.5% on the 11th month.
